# Indents

**Published:** 1901-04-15

**Authors:** 


### INDENTS.

☞ **Brief Articles of Technical or Scientific Value will receive notice and comment. Contributions invited.**

FOR CHAPPED HANDS AND LIPS.—This formula is about as nearly *perfect* as could be wished for: Glycerin 2 oz., Aq. dist. 2 oz., Ol. cajuputi xxx gtts. Perfume q. s. M.—W. G. Steele, *Medical Record.*

FINISHING GUTTA PERCHA FILLINGS.—Hot vaseline is a solvent of gutta percha, and is useful in trimming gutta percha fillings. Apply the vaseline and use a warmed burnisher.—L. Van Orden, *Items of Interest.*

SOLDER ALLOY.—Four parts silver C. P.; two parts copper C. P.; one and one-sixth parts zinc C. P. To make the solder, add one part of the alloy and three parts of coin gold.—Dr. R. Wakefield, in *Items of Interest.*

IN USING THE MALLET.—If you would have your patient thank you, use a piece of cork, trimmed square, between the teeth when inserting a gold filling. The impact of the mallet blows is less severe on a tooth thus supported by a cushion of cork.—*Dental Hints.*

GERMICIDE; ACETIC ACID.—The investigations of Drs. Abbott and McCormick, of the Johns Hopkins University, show that a solution of bichlorid of mercury containing 7 percent of acetic acid is more effective as a germicide than bichlorid of mercury alone.—*Items of Interest.*

COMPRESSED AIR IN BLEACHING TEETH.—In bleaching teeth I find that by the application of hot air at high pressure I am able to produce the required conditions in one-half the usual time, rapidly evaporating 25 percent pyrozone and forcing it into the tubuli.—S. Freeman, *International Dental Journal.*

SUPRARENAL CAPSULE.—Dr. E. H. Raymond uses this agent as a local styptic for hemorrhage from congested gums, applies the powder directly to the soft or spongy gums. It checks the bleeding at once and leaves no soreness or excoriation. It is harmless and odorless.—New York letter in *Dental Review* for February.

TANNOCREOSOFORM.—This is a combination of tannin, creosote and formaldehyd, and is a brown, odorless and tasteless non-toxic powder, insoluble in water and glycerine, but soluble in dilute alkalies. It is an active antiseptic, suitable for both internal and external use. As much as five grammes may be given daily.—*Nouveaux Remedes.*

MOLAR TOOTH IMPLANTED IN THE ORBITAL PLATE OF THE MAXILLARY SINUS.—M. G. Liaras reports such a case, the tooth being found in the orbital plate of the left maxillary sinus. A sinusitis developed, which yielded promptly to surgical measures. The sinus remained open and was treated antiseptically. The tooth was not removed.—*Revue hebdomadaire de laryngologie, d'otologie et de rhinologie*,

COLLODION AS A MODEL VARNISH.—Dr. D. H. Payne in the *Items of Interest* recommends collodion as a better varnish than sandarach, for varnishing plaster models previous to packing the rubber for vulcanizing. It does not adhere to the vulcanized plate and leaves a smooth and hard surface. To separate model from plaster impression varnish with thin collodion and dust the surface with talcum powder, brushing away all loose powder.

Antidote for Cocain.—Dr. C. Grove in *Medical Visitor* recommends gelsemium as an antidotal resuscitant in cocain collapse from injection of cocain for dental purposes, resulting in syncope, etc. [Its action in such cases is rather obscure from its known physiological action. It is probably due to its stimulating action on the respiration and supporting action to the heart ganglia, together with the fact that it stimulates the vaso motor system and thus restores the normal blood pressure.—Ed.]

Copper Amalgam as a Filling for Deciduous Teeth.—Dr. J. J. Burke, in *Dental Cosmos*, advocates the use of copper amalgam as a filling material for deciduous teeth because it prevents recurrence of decay, is easily manipulated under circumstances which would prove fatal to most other filling materials, and it is not inimical to the sensible conditions of tooth tissues, and withall is sufficiently durable to serve until the teeth are replaced by the permanent. Its color contra-indicates its use in the anterior teeth.

Alveolar Abscess with Necrosis.—A patient twenty-five years of age had an alveolar abscess with fistula on left upper lateral, which resisted escharotic and disinfectant treatment made through the root. I enlarged the fistula to permit clear view and instrumentation and then excavated all necrotic tissue until the sensitive tissue was reached. Then excised and smoothed the end of the root, and packed the cavity with a solution of boracic acid on cotton. The cavity was irrigated every day for a week with boracic acid solution, and then every other day for a week more. In two weeks the wound healed.—R. C. Smith, D.D.S., in *Dental Review*.

Setting Banded Crowns.—Dr. Taggart says: "Set all banded crowns and bridges with pink base plate gutta-percha. Why? First, because it acts as a cushion between the crown and root, and if any undue strain comes it will yield before it will break. It acts to cushion the blow. Second, you can be more uniformly successful, as no haste is necessary, and third, as no haste is necessary, it takes away nearly all of that nerve strain which always accompanies the setting of a crown or bridge with cement. Fourth, the crown or bridge can be taken off at any time within five minutes for repairs or to fill an adjoining tooth; certainly a sufficient number ot reasons for at least trying the process, which, if successfully mastered, will wean you forever from setting crowns or bridges with cement.

